# Repeating themes of plastic genes and therapeutic schemes targeting the ‘tandem repeatome’

**DOI:** 10.1093/braincomms/fcae047

**Published:** 2024-02-19

**Authors:** Anthony J Hannan

**Affiliations:** Florey Institute of Neuroscience and Mental Health, University of Melbourne, Parkville, Australia; Department of Anatomy and Physiology, University of Melbourne, Parkville, Australia

## Abstract

This scientific commentary refers to ‘Modification of Huntington’s disease by short tandem repeats’ by Hong *et al*. (https://doi.org/10.1093/braincomms/fcae016) in *Brain Communications*

**This scientific commentary refers to ‘Modification of Huntington’s disease by short tandem repeats’ by Hong *et al*. (** https://doi.org/10.1093/braincomms/fcae016 **) in *Brain Communications***

Human disorders are often, somewhat simplistically perhaps, described as monogenic (e.g. Huntington’s disease and fragile X syndrome) or polygenic (e.g. major depressive disorders and common forms of dementia such as Alzheimer’s disease). However, the reality of clinical genetics and genomics is not nearly this simple. A prime example of monogenic disorders is the group of over 60 (and still expanding) tandem-repeat disorders, resulting from mutations in over 60 (out of ∼2 million) of the tandem repeats distributed across the human genome.^1^ Huntington’s disease is a classic example of such a tandem-repeat disorder, and is caused by a cytosine-adenine-guanine trinucleotide (CAG) tandem-repeat expansion in the *huntingtin* (*HTT*) gene, encoding an extended polyglutamine tract in the huntingtin protein. Huntington’s disease is one of the more common monogenic tandem-repeat disorders, and involves a devastating combination of cognitive, psychiatric and motor symptoms (as well as peripheral symptoms such as gastrointestinal dysfunction and dysbiosis), with neurodegeneration progressing to cause death within one or two decades (and faster in ∼5% of cases exhibiting juvenile onset). However, it has been shown, using the genome-wide association study (GWAS) approach, that other genes, and their polymorphic variance, can modulate the onset (and progression) of Huntington’s disease.^2^ Thus, while the tandem-repeat expansion in the CAG trinucleotide repeat of HTT is the primary driver of Huntington’s disease pathogenesis, a variety of genetic modifiers, as well as environmental modifiers, can affect onset of symptoms and disease progression.

A new study by Hong *et al.*,^3^ in their article in this issue of *Brain Communications*, has demonstrated that polymorphic variance in other short tandem repeats (STRs), outside of the CAG-repeat STR in the *HTT* gene and in many other parts of the genome, can act as disease modifiers with respect to Huntington’s disease pathogenesis. The authors first investigated whether the CAG tandem repeats in any of the other genes causing polyglutamine diseases (Huntington’s disease is the most common of at least nine polyglutamine diseases) could act as genetic modifiers of age-at-onset of Huntington’s disease. The investigators obtained negative results in this first part of their study.^3^

However, in the next part of their investigation, these investigators performed imputation using single-nucleotide polymorphism (SNP) microchip haplotypes from GWAS data.^3^ This imputation approach uses prior data sets, along with the GWAS SNP data, to impute approximate STR repeat lengths (genome wide). Hong *et al.*^3^ found a striking result that repeat-length polymorphisms in genome-wide STRs can act as genetic modifiers of age-at-onset of Huntington’s disease.

Hong *et al.*^3^ identified significant genetic modifiers tagged by STRs on Chromosomes 2, 8 and 15. These could be linked to the genes *PMS1*, *RRM2B* and *FAN1*, identified via traditional SNP-based GWAS approaches (without imputation of STR repeat lengths) in prior research.^2^ Similarly, the MLH1 region of Chromosome 3 and the MSH3 region of Chromosome 5 (both linked via SNP-based GWAS) showed associations with STR repeat lengths.^3^ Furthermore, the authors reported a near-significant association on Chromosome 15q linked to a guanine-adenine dinucleotide (GA) tandem repeat in the synaptic vesicle glycoprotein 2B (*SV2B*) gene. The authors noted that direct repeat genotyping was still required to validate the potential roles of such genetic modifiers in Huntington’s disease pathogenesis.^3^ Nevertheless, this evidence for tandem-repeat polymorphisms in STRs as genetic modifiers in Huntington’s disease is tantalizing, and has major therapeutic implications.

These new findings need to be interpreted in light of the previous proposal that tandem repeats (including STRs as well as variable-number tandem repeats) may help explain the ‘missing heritability’ resulting from previous GWAS investigations of polygenic diseases and traits.^4^ Since this original proposal in 2010,^4^ extensive evidence has emerged to show that tandem-repeat mutations and tandem-repeat polymorphisms contribute to a variety of common human conditions. Some of the most striking evidence, of relevance to the present article, comes from the autism field.^5-7^ However, more recent studies also implicate genome-wide tandem repeats in schizophrenia^8,9^ and Parkinson’s disease,^10^ among other common polygenic disorders. One caveat of the new study by Hong *et al.*,^3^ as noted by these investigators, is that they only imputed STRs. Future approaches should consider all of the tandem repeats in the human genome, including all STRs (with repeat motifs of 1–6 nucleotides) and variable-number tandem repeats (with longer repeat motifs).

These and other findings raise important questions regarding the complex and almost entirely unexplored roles of ∼2 million tandem repeats, which collectively occupy around 8% of the human genome. Furthermore, a total of ∼50% of the human genome consists of repeatome, including not only all of these tandem repeats but also other repetitive DNA such as copy-number variants and transposable elements.^1^ While the *HTT* gene and the other 60-odd genes that have been implicated in monogenic tandem-repeat disorders have been explored in some detail, this leaves almost 2 million human tandem repeats whose structure and function (including the subset of transcribed RNAs and translated proteins), and contribution to human biology and disease, remains largely a mystery.

This new study^3^ also extends our understanding of Huntington’s disease pathogenesis, allowing us to more fully comprehend pathogenic mediators and genetic and environmental modifiers, as well as potential gene–gene and gene–environment interactions (Fig. 1). Figure 1 attempts to convey some of the complexities of the ‘tandem repeatome’ (the subset of the repeatome consisting of tandem repeats), with respect to genome-wide tandem repeats, and the way in which tandem-repeat polymorphisms can modulate structure and function at the levels of the genome (repeat-containing DNA in genic and intergenic regions), transcriptome (repeat-containing RNA transcripts) and proteome (proteins containing amino-acid repeats).

**Figure 1:**
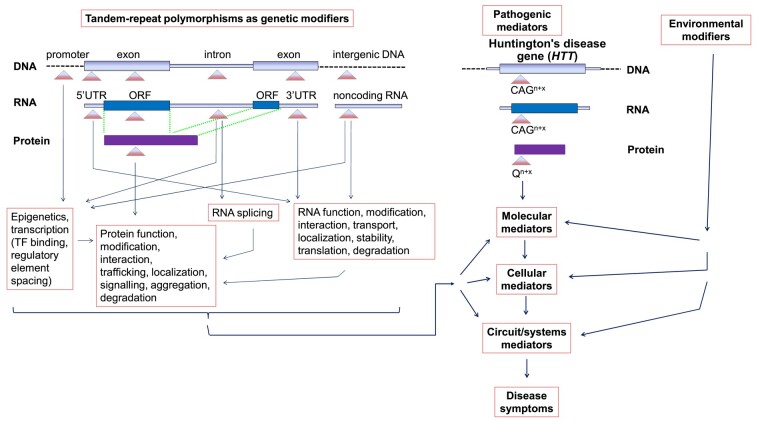
**A schematic illustrating how tandem-repeat polymorphisms can act as genetic modifiers in Huntington’s disease, in concert with pathogenic mediators and environmental modifiers.** In the middle of the figure, pathogenic mediators of Huntington’s disease, from the CAG tandem repeat in the *huntingtin* (*HTT*) gene onwards, are illustrated (the exon/intron structure of the *HTT* gene is not shown). The CAG-repeat number is expanded (*n* + *x*) in Huntington’s disease, and once transcribed into mRNA and translated into huntingtin protein, with the number of glutamine (Q) amino acids also expanded (*n* + *x*), it leads to a cascade of molecular, cellular, circuit and systems mediators and an eventual onset of disease symptoms. This new study by Hong *et al.*^3^ demonstrates that STRs and their associated tandem-repeat polymorphisms can act as genetic modifiers of Huntington’s disease. Depending on the location of tandem repeats across the genome, as well as the nature of the repeat motifs and repeat numbers, they can have a variety of effects on the structure and function of DNA and the many RNAs and proteins transcribed and translated from tandem-repeat–containing regions.^1^ Collectively, these tandem-repeat polymorphisms can act as genetic modifiers, at multiple levels of the pathogenic pathway. Of course, environmental modifiers were shown to play a role in Huntington’s disease over two decades ago, and they may combine with the genetic modifiers via complex gene–environment interactions. Each triangle represents one allelic range of tandem repeats (of ∼2 million) in the human genome, with the upper tip of the triangle representing the shortest repeat length and the upper base of the triangle representing the longest repeat length, across a polymorphic range that can vary between individuals, and sometimes between cells and tissues in an individual, via somatic (including developmental) tandem-repeat mutation. CAG*^n^*  ^+^  ^*x*^, number range (*n* + *x*) of CAG repeats causing disease; HTT, huntingtin; ORF, openreading frame; Q, glutamine; Q*^n^*  ^+^  ^*x*^, number range (*n* + *x*) of glutamine repeats causing disease; UTR includes 5′UTR and 3′ UTR.

Tandem-repeat polymorphisms could thus act as genetic modifiers of Huntington’s disease in a multitude of ways. This could include, for example, modulating the epigenetic regulation and transcription of modifier genes encoding proteins that in turn modulate molecular and cellular mechanisms of pathogenesis (Fig. 1). Alternatively, a tandem-repeat polymorphism encoding a repetitive tract of amino acids in the protein product of a genetic modifier could act directly on the structure and function of that protein to modulate the molecular aspects of pathogenesis and thus the rate (either positively or negatively) of disease onset and progression (Fig. 1).

Importantly, such new information regarding molecular mechanisms associated with genetic modifiers could be used to develop novel therapeutic approaches. In the case of Huntington’s disease, the identification of additional STR genetic modifiers could have direct therapeutic implications via candidate target identification. A genetic modifier that either accelerates or decelerates age at disease onset could potentially be ‘tweaked’ therapeutically, in the appropriate direction, to delay disease onset and slow progression.

A molecular modifier that accelerated pathogenesis could be targeted and inhibited with a new therapeutic agent. Conversely, a molecular modifier that decelerated pathogenesis could be therapeutically activated or mimicked, and this could also provide a new candidate drug to be tested in pre-clinical and clinical trials. It is therefore an urgent priority, as there are no disease-modifying therapies currently available for Huntington’s disease, to pursue these newly discovered genetic modifiers as potential therapeutic targets.

In attempting to understand how and why tandem repeats have evolved and populated almost every ‘nook and cranny’ of the human genome, we must consider some of their unique attributes. Tandem repeats can enhance ‘genetic plasticity’, as they are often far more mutable (during meiosis, mitosis and post-mitotically) than single nucleotides and other non-repetitive sequences.^1,4^ Thus, tandem-repeat–containing genes might be considered to be more plastic than genes without tandem repeats. In meiosis and associated germ lines, this could provide enhanced genetic variability on which natural selection might act. In mitosis, during development and at organismal maturity, this could lead to cellular selection within an organism.^1,4^

One key lesson from this study from Hong *et al.*^3^ is that GWAS conducted purely using SNP-based microchips, without imputation of tandem-repeat polymorphisms, is potentially missing crucial information.^4^ Tandem repeats and other components of the repeatome are not ‘junk DNA’ but rather selectively evolved sequences (as demonstrated by comparative and evolutionary genomics) whose structures and functions (at the levels of genomes, transcriptomes and proteomes) we must urgently try to understand. Utilizing the latest technologies (e.g. long-read sequencing with PacBio and Oxford Nanopore Technologies) and bioinformatic approaches, tandem-repeat modifiers of other monogenic disorders can also be discovered, using GWAS and other genome-wide approaches.^11-13^ Furthermore, we are only at the very beginning of understanding how tandem repeats contribute to the pathogenesis of a wider range of common polygenic disorders. The recent progress with autism,^5-7^ schizophrenia^8,9^ and Parkinson’s disease^10^ suggests that tandem-repeat GWAS should be systematically investigated for other neurological and psychiatric disorders. This could include amyotrophic lateral sclerosis (ALS), the most common form of motor neuron disease (MND), where the C9ORF72 hexanucleotide tandem repeat provides a striking exemplar (and also contributes to frontotemporal dementia^1^), Alzheimer’s disease, multiple sclerosis, depression and bipolar disorders, to name a few. Furthermore, tandem repeats are also being increasingly implicated in other human disorders, such as developmental disorders and many cancers,^14,15^ as well as biological traits.

In summary, this latest study by Hong *et al.*^3^ provides an important new piece in the puzzle of Huntington’s disease pathogenesis, and thus has the potential to advance therapeutic development for this deadly disease, for which there are currently no disease-modifying therapies available. However, this study has broader lessons for the scientific community exploring clinical genetics, genome-wide genetic modifiers, GWAS and monogenic and polygenic disorders more generally. While SNPs have naturally been the focus of the last two decades of GWAS investigations (due at least partly to the technical ease of SNP genotyping on microchips), whether in search of genetic modifiers or causal variants, the repeatome (which, I repeat, constitutes around half of the human genome!) has been largely overlooked. It is time that we focused more on this ‘genomic dark matter’ of the repeatome, including the ‘tandem repeatome’, in genome-wide searches for genetic modifiers and causal variants. This will enhance our capacity to prevent, treat and eventually cure a range of devastating disorders of the brain and body.
